# Empty Seeds Are Not Always Bad: Simultaneous Effect of Seed Emptiness and Masting on Animal Seed Predation

**DOI:** 10.1371/journal.pone.0065573

**Published:** 2013-06-11

**Authors:** Ramón Perea, Martin Venturas, Luis Gil

**Affiliations:** 1 Departamento de Silvopascicultura, ETSI, Montes, Universidad Politécnica de Madrid, Ciudad Universitaria, Madrid, Spain; University of Marburg, Germany

## Abstract

Seed masting and production of empty seeds have often been considered independently as different strategies to reduce seed predation by animals. Here, we integrate both phenomena within the whole assemblage of seed predators (both pre and post-dispersal) and in two contrasting microsites (open vs. sheltered) to improve our understanding of the factors controlling seed predation in a wind-dispersed tree (*Ulmus laevis*). In years with larger crop sizes more avian seed predators were attracted with an increase in the proportion of full seeds predated on the ground. However, for abundant crops, the presence of empty seeds decreased the proportion of full seeds predated. Empty seeds remained for a very long period in the tree, making location of full seeds more difficult for pre-dispersal predators and expanding the overall seed drop period at a very low cost (in dry biomass and allocation of C, N and P). Parthenocarpy (non-fertilized seeds) was the main cause of seed emptiness whereas seed abortion was produced in low quantity. These aborted seeds fell prematurely and, thus, could not work as deceptive seeds. A proportion of 50% empty seeds significantly reduced ground seed predation by 26%. However, a high rate of parthenocarpy (beyond 50% empty seeds) did not significantly reduce seed predation in comparison to 50% empty seeds. We also found a high variability and unpredictability in the production of empty seeds, both at tree and population level, making predator deception more effective. Open areas were especially important to facilitate seed survival since rodents (the main post-dispersal predators) consumed seeds mostly under shrub cover. In elm trees parthenocarpy is a common event that might work as an adaptive strategy to reduce seed predation. Masting *per se* did not apparently reduce the overall proportion of seeds predated in this wind-dispersed tree, but kept great numbers of seeds unconsumed.

## Introduction

Seed predation by granivorous vertebrates is a common fate of seeds and may represent a significant loss of viable seeds, reducing plant reproduction efficiency [1], [2]. Seed predation is also considered an important selective pressure which drives the evolution of seed characteristics [3]. Many different mechanisms have been reported to reduce seed predation by animals. Among them, chemical (e.g. secondary compounds) and physical seed properties (e.g. seed size and coat hardness) are the most common and conspicuous forms of defense against seed predators [4]. However, seeds from a particular plant species are usually consumed by many different guilds of seed predators (e.g. insects, birds, mammals), which differ in body size, gut characteristics, temporal and spatial scales, and ability to cope with such plant defenses [4]–[6]. Consequently, some seed attributes may function against certain seed predators but not against others. In many cases, poor knowledge of the multiple species involved in the predation of seeds of a particular plant species hinders the understanding of plant-granivore coevolution.

Seed foragers benefit from plants producing large seed crops as it increases their energy intake and reduces their effort and time costs, whereas plants pay a higher cost of reproduction [7], [8]. In addition, full seeds (those containing an embryo) are more nutritious items than empty seeds and, therefore, preferred by foragers [9], [10]. Hence, the handling cost required by a seed predator to find a valuable seed increases as the proportion of full seeds decreases [12], [13]. Multiple studies have documented that production of empty-seeded fruits can reduce pre-dispersal seed predation by insects [14]–[17] or even by vertebrates [12], [13], [18]. However, reductions in seed predation are usually calculated as a proportion of the total seed crop (including both empty and full seeds) and not as a proportion of the full seeds that escape predation [18]. This approach does not completely assess the real effect of masting and seed emptiness on the reproductive success. Although very few investigations have addressed the correlation between crop size (or masting) and the proportion of empty seeds [13], [18], most of them have neglected to explore the simultaneous action of seed masting and the proportion of empty seeds on the rate of full seed consumption by animals.

Most studies that have focused on seed emptiness as a mechanism to reduce seed predation have only accounted for pre-dispersal seed predation, and not for subsequent possible predations (post-dispersal) which are, in many plant species, quantitatively more important [19]–[21], especially in wind-dispersed plants [22]. In fact, most wind-dispersed seeds are not predated in the tree and fall onto the ground among different microsites [23]. Since microsite of seed deposition represents a significant factor in seed encounter and predation by ground foragers [24], [25], we should not overlook the importance of empty seeds (at variable proportions) in different microsites, because it may influence the overall seed predation and seedling establishment. Only by addressing the whole assemblage of predators (both pre and post-dispersal) and their foraging behaviour (including microsite effect) can we understand the real effect of empty seeds on the predation of full seeds and the possible ecological implications for plant regeneration.

Despite the fact that many genera of wind-dispersed trees produce empty seeds and are widely distributed taxonomically (e.g., *Ulmus*, *Acer*, *Pinus*, *Fraxinus, Salix*), these trees have received very little attention in relation to the evolutionary and ecological consequences of producing empty-seeded fruits. However, wind-dispersed plants are crucial to understanding hypothetical adaptations to seed predation because, unlike animal-dispersed plants (extensively studied), in wind-dispersed plants, animals are not required for successful seed dispersal and so they act mainly as seed predators. In this respect, it would be interesting to estimate the minimum proportion of empty seeds necessary to succesfully deceive the predators and, simultaneously, maximize seed survival.

Our hypothesis is that empty seeds might contribute to overall plant fitness by increasing the proportion of full seeds that will escape predation both pre and post-dispersal. Likewise, we hypothesize that high crop size (either full or empty-dominated seed crop) will also reduce animal seed predation following the predator satiation hypothesis [14], [26]. We tested these two hypotheses using an elm tree (*Ulmus laevis* Pall.) as an example, since its fruits (samaras) are dispersed by wind and water (not by animals). Here, we carry out this analysis within the whole assemblage of seed predators (both pre and post-dispersal) and in two contrasting microsites (open vs. sheltered), in order to evaluate their effect on plant reproductive success.

## Methods

### Ethics Statement

All the work was conducted in accordance with relevant national and international guidelines, and conforms to the legal requirements of the Regional Government (Madrid, Spain) and Public Administration. Regional Government of Madrid gave us permission to conduct the study in this site. Field studies did not involve endangered or protected species. Animals were only observed in the field, neither captured nor harmed.

### Study Area and Species

This study was conducted in a riparian forest in Madrid province, Central Spain (40°32′ N, 3°40′ W). The riparian forest is located within a 330 ha public domain forest, at 700 m a.s.l. and in a Mediterranean climate [annual precipitation of 426±124 mm (years 1973–2011) with a 3-month summer dry period]. The riparian forest is composed mainly of elms (*Ulmus laevis* Pall.) and a few ashes (*Fraxinus angustifolia* Vahl.) and willows (*Salix salviifolia* Brot. and *S. atrocinerea* Brot.). The understory is a mosaic of evergreen shrubs (mainly *Rubus ulmifolius* Schott.), tall forbs and grasses. The site contains 53 mature elms (d.b.h.>10 cm) and 104 saplings spread along a small stream.

*U. laevis* is mainly found in Central and Eastern Europe. In the southernmost part of its distribution (Spain) the populations are small and rare [27]. Like other elm species, *U. laevis* flowers before leaf bud breaks and its fruits are winged nuts (samaras) with a single seed. Natural seed fall of *U. laevis* occurs in April-June. Elm samaras can fall onto the ground *in situ* or be carried by wind or water [28]. Seeds of other European elms (e.g. *U. minor* Mill.) are known to be preyed upon by vertebrates (mainly rodents) after seed dispersal [29].

**Figure 1:**
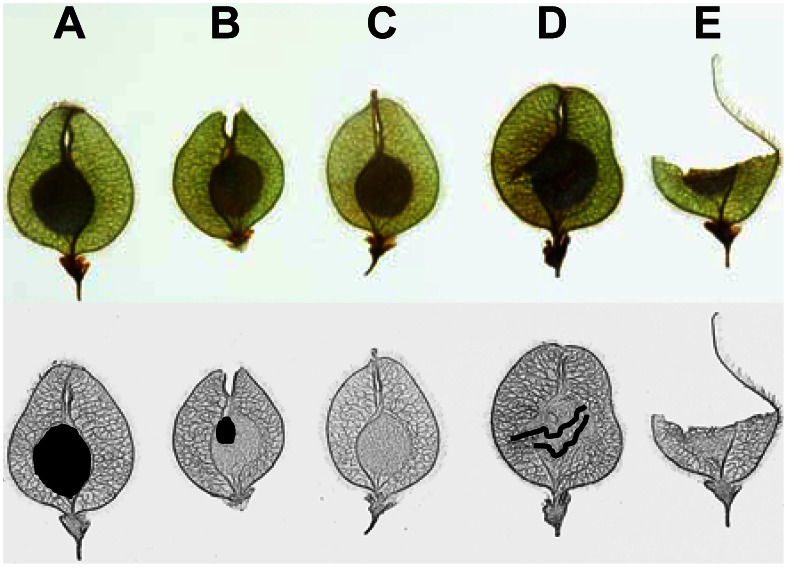
*Ulmus laevis* samara characterization. Pictures of the different samara categories are shown on the top row and sketches on the bottom row: (A) full samara (FS); (B) undeveloped samara (US); (C) empty samara (ES); (D and E) predated samaras (PS). The black colour of the sketch stands out the seed for FS and US, and the tissues torn by animals in the PS.

We found four types of elm samaras which were easily distinguished by their morphological characterization (Fig. 1): (a) *Full samaras* (FS), fruits properly developed with a full seed in the centre; (b) *Undeveloped samaras* (US), fruits with an undeveloped seed in the centre. This seed does not develop because the embryo aborts in the early stages of development. The death of the embryo can be due to environmental conditions (strong frosts) or to deleterious genes [30]; (c) *Empty samaras* (ES), fruits that develop with no seed. These are generally formed by parthenocarpy, that is, without flower pollination [27]; (d) *Predated samaras* (PS), full samaras wherein seeds have been eaten by animals.

### Samara Production

We placed 20 seed traps (1 m×1 m) on the ground during three consecutive *Ulmus* seed dispersal seasons (April-June, 2009–2011). Traps were located randomly along 425 m of the riparian forest and no protection was used to prevent access by animals. We collected all samaras and counted them every 2–3 days during the dispersal season. We divided the counted samaras into the four possible categories (see above and Fig. 1). However, for evaluating seed production, we considered predated samaras as full samaras.

To evaluate possible inter-individual differences in the production of full, undeveloped and empty samaras, we randomly selected 35 and 19 elm trees in years 2009 and 2010, respectively. We then collected samaras from the tree branches (from 3 to 5 dm^3^ of samaras per tree). From each sample, we extracted 400 samaras and counted the number of samaras in each category. Finally, to assess the energy cost of producing empty samaras (ES) and full samaras (FS) we took four groups of 100 samaras of each one of these types. We did not take individual samaras because each empty samara weighs very little to be individually weighed with accuracy. Then we weighed them after five days of oven drying at 60°C to compare their dry biomass. In addition, ES and FS samples were sent to a chemical laboratory (three samples per type) to obtain their relative cost in allocation of nutrients, especially N and P, which are important elements in seeds [31]. Each sample contained 1 g of dry pulverized samaras (equivalent to *ca.* 254 ES and *ca.* 122 FS, respectively).

### Identification of Seed Predators

In order to identify the seed predators, four motion-detection digital video cameras with night vision were used (Leaf River IR-5, 5 MP). Cameras were placed beneath elm trees (where most seeds were located) at 1.0–1.5 m height, pointing at the ground. We avoided shrub cover and tall grasses within the field of view of the cameras to easily record the animals consuming seeds. Cameras were moved (distance >50 m) every 35–60 days, and were used in April-June, coinciding with the elm seed dispersal period, in two consecutive years (2010 and 2011). We unsuccessfully placed cameras in the trees (2–3 m height) to record possible arboreal seed predators during three weeks in 2010, but very few recordings of potential seed predators were obtained. We only considered those recordings that contained animals consuming elm seeds.

### Seed Predation in the Tree

We selected three observatories within the study area. Observatories were three locations separated at least 90 m from each other where one person could easily watch the animals consuming elm seeds. In 2010 we spent 1602 minutes observing the foraging behavior of birds and squirrels up in the elm trees. We used binoculars (8×42), spotting scopes (20–60×65) and stopwatches to estimate the number of elm seeds each bird preyed upon. Observations were mostly performed in the mornings (8.00–10.30 am) and evenings (5.30–8.00 pm), and always during the fruit ripening season (April-June 2010 and 2011). In 2011 due to the extreme lack of seeds eaten by seed predators (low crop size) we only spent 540 minutes observing them.

### Seed Predation on the Ground

**Figure 2:**
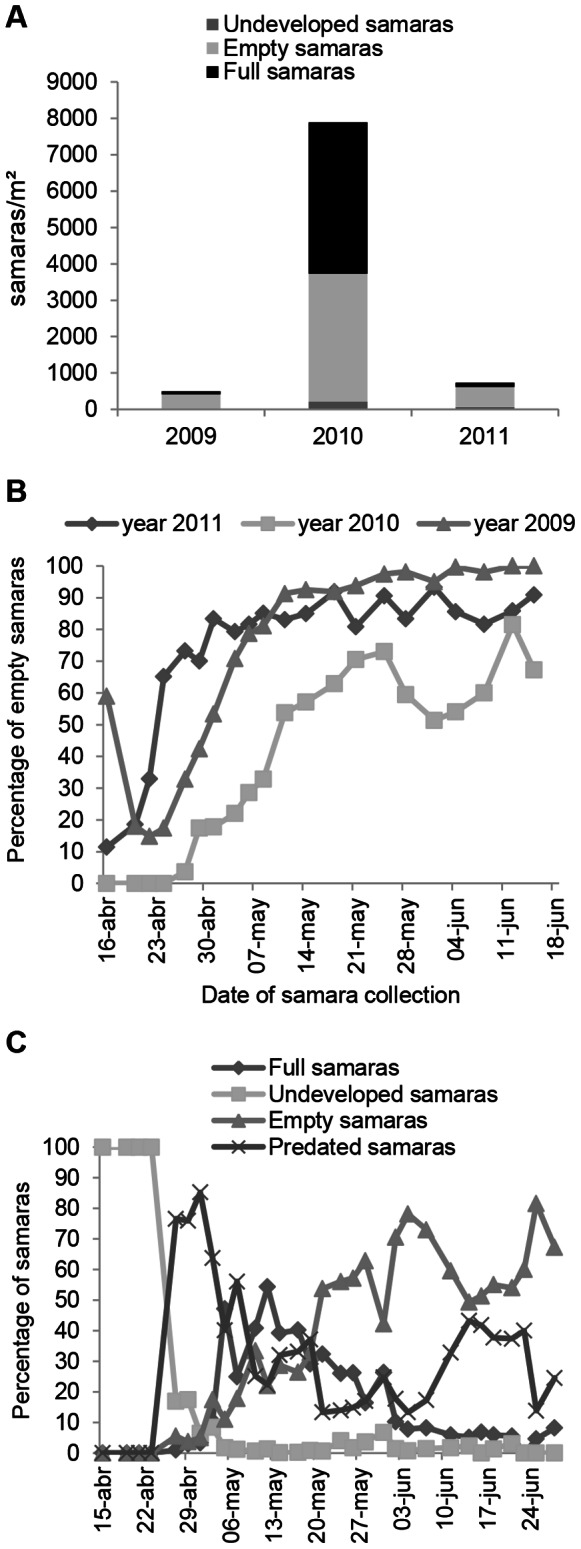
Samara production. a) Fruit crop size for each samara category and season; b) proportion of empty samaras dispersed throughout each season; c) proportion of each samara category dispersed along the mast year 2010.

We selected 15 sample points within the study area. Sample points were located at approximately 25–30 m from each other. In each sample point two microhabitats were distinguished: sheltered (dense shrub cover) and open (only grasses). Each microhabitat contained three types of depots (90 depots in total): (1) invertebrate access only, built with a wire mesh in a cubic shape (15 cm×15 cm×15 cm) and buried into the soil (5 cm approximately); (2) rodent and invertebrate access, made of a mesh (50 cm×50 cm) placed 3 cm above the ground surface with open sides, and (3) all seed foragers access, with no exclosure (this includes e.g. birds and medium-large mammals). In each depot we placed one Petri dish (9 cm diameter) containing 60 *Ulmus laevis* samaras. Depots remained in the same place throughout the experiments. We conducted 6 trials in two consecutive years (2010–2011) according to the natural samara availability for the ground seed predators. Natural samara availability refers to the number of samaras that were found naturally on the ground (low vs. high) at the time of the seed offer, based on the data obtained from the seed traps (Fig. 2). We considered both intra and inter-annual seed availability. High availability was only considered for the late season of the mast period, when great amounts of seeds were found on the ground (June-July 2010). Thus, in 2010 two trials were performed under high seed availability (June and July; seed density >5500 seeds m^−2^) and two under low seed availability (April and late September; seed density <200 seeds m^−2^). In 2011 only two trials were performed (June and September), both with low seed availability due to the low seed crop (seed density <300 seeds m^−2^). Because ES are not always available (e.g. in the early season) we used FS (collected in previous years) in three trials, two with low (April 2010 and June 2011) and one with high seed availability (July 2010). Thus, to avoid unbalanced experiments, we placed both ES and FS in the other three trials, also two with low (September 2010 and September 2011) and one with high seed availability (June 2010). In this latter experiment, one third of the Petri dishes contained 10% FS (90% ES), another third contained 50% FS (50% ES) and the other third contained only FS (0% ES). The percentage of FS per Petri dish in each trial was randomly assigned. A total of 32,400 samaras (90 depots×6 trials×60 seeds) were offered to seed foragers. We checked the depots every 2–3 days during six visits (until the day 14th–20th after seed offer). In year 2011we stopped checking the depots if no changes were found after two consecutive visits. In each visit, we noted whether samaras were predated *in situ* or removed.

### Bird Density Estimates

To estimate the abundance of avian seed predators we designed a permanent linear transect in the study area (470 m in length and approximately 50 m in width). Surveys were performed weekly during the seed ripening period (from late April to early June) in two consecutive years (2010 and 2011). Bird censing started at approximately 8∶30–9∶00 am and we attempted to avoid heavy rain, poor visibility or strong wind conditions. Following the bird survey instructions from the British Trust for Ornithology (www.bto.org) we recorded all birds we saw and heard.

### Data Analysis

We used one-way ANOVA to analyze the possible chemical differences in the allocation of nutrients (C, N, P, Fe, K) between ES and FS. To analyze seed predation on the ground, we performed Generalized Linear Mixed Models (GLMM). The response variable was the proportion of samaras predated in each depot at each trial in relation to the number of full seeds offered (proportional response variable; binomial error family; Total number of observations = 540). The fixed effects (factors) considered in the GLMM were microhabitat of samara deposition (shrub vs. open), guild of foragers involved (depot type), samara quality (percentage of full samaras in the depot; three levels) and natural samara availability (low vs. high). Those trials with only FS were also included in the model. We also considered the two-way interactions among the fixed effects. Sample point was included as a random effect. The model was fitted by the Laplace approximation according to our data properties [32] using the “lmer” function within the “lme4” package for R 2.13.1 software (www.r-project.org). We checked for overdispersion (considering the complete range of degrees of freedom for the random effect; [32]). We computed all possible models by using the function “dredge” in the package “MuMIn”. We only selected the best supported model (ΔAIC = 0; Akaike weight = 0.69) since the next best-fitting model showed ΔAIC>2, giving substantial evidence to the selected model [33]. For the statistical inference of the main effects we performed a Wald χ^2^ test with the selected model, following the decision tree for GLMM fitting and inference (<3 random effects; no overdispersion; no inference interest for the random effect; [32]). To ensure that our inferences were not biased and, thus, avoid possible spurious correlations, we used a randomization approach with bootstrap. We resampled the response variable (2000 times; N = 540 random samples) by random selection with replacement (bootstrapping). We, then, obtained the 95% confidence intervals of each model parameter and compared them with the observed confidence intervals of the selected model. We also randomized the predictors “seed availability” and “seed quality” (2000 times each) to ensure that there were no spurious correlations between the response variable (seed predation) and the two predictors. In both cases we used random samples (N = 540) with replacement.

Finally, estimation of the density of each bird species was obtained following [34] where all existing individuals were assumed to be recorded within the transect limits. Two-tailed t-tests for unequal variance were used to compare density estimates of each bird species during the two years.

## Results

### Samara Production

In 2010, *U. laevis* seed crop was extremely high (7891 seeds m^−2^), whereas in 2009 and 2011 samara production was less than 10% that of 2010 (Fig. 2A). We found the highest proportion of empty samaras in 2011 (76.1%), the lowest for the mast 2010 (44.6%) and an intermediate value for 2009 (70.5%; Fig. 2A). Thus, in 2009 and 2011 full samaras (FS) only represented 17.9% and 15.4% of the crop, respectively, whereas in 2010 (the mast year) 52.7% of fruits were FS (Fig. 2A). However, in the mast 2010 we collected the highest proportion of predated seeds (23.1%; *n* = 1824 seeds m^−2^), followed by 2009 (17.2%; *n* = 87 seeds m^−2^) and 2011 (13.6%; *n* = 100 seeds m^−2^). Surprisingly, we found a similar pattern in the dispersal of empty samaras throughout the three seasons, regardless of the crop size, with an increasing fall of empty samaras along the seed drop season (Fig. 2B). Additionally, we found no inter-annual differences in the synchronization of the FS fall (i.e. period of time elapsed to drop 90% of the FS crop). Thus, synchronization of the FS drop only varied from 21 to 28 days among the three years. This period for the FS drop was approximately 2–3 times shorter than the whole period of seed drop (including all types of samaras; Fig. 2C). Undeveloped samaras fell early in the season (mostly in the first two weeks; Fig. 2C) and represented a very low proportion of the seed crop (Fig. 2A).

We found a higher inter-individual variability in the proportion of empty samaras in the mast 2010 (mean±SD = 25±24%; range 3–95%; CV = 0.98; *n* = 19 trees) than in the low crop 2009 (mean±SD = 57±26%; range 9–98%; CV = 0.46; *n* = 35 trees). Individuals also showed a high intra-individual variability in their production of empty samaras between both years (Mean CV = 0.60; *n* = 11 trees). Dry biomass of empty samaras was less than half (378±10 mg per 100 seeds) of those full (820±11 mg per 100 seeds). In addition, full samaras showed a 13%, 27% and 128% higher C, P and N content than empty samaras, respectively (*P*<0.05). However, empty samaras had a 21% and 22% higher content of K and Fe, respectively (*P*<0.05).

### Identification of Seed Predators

**Figure 3:**
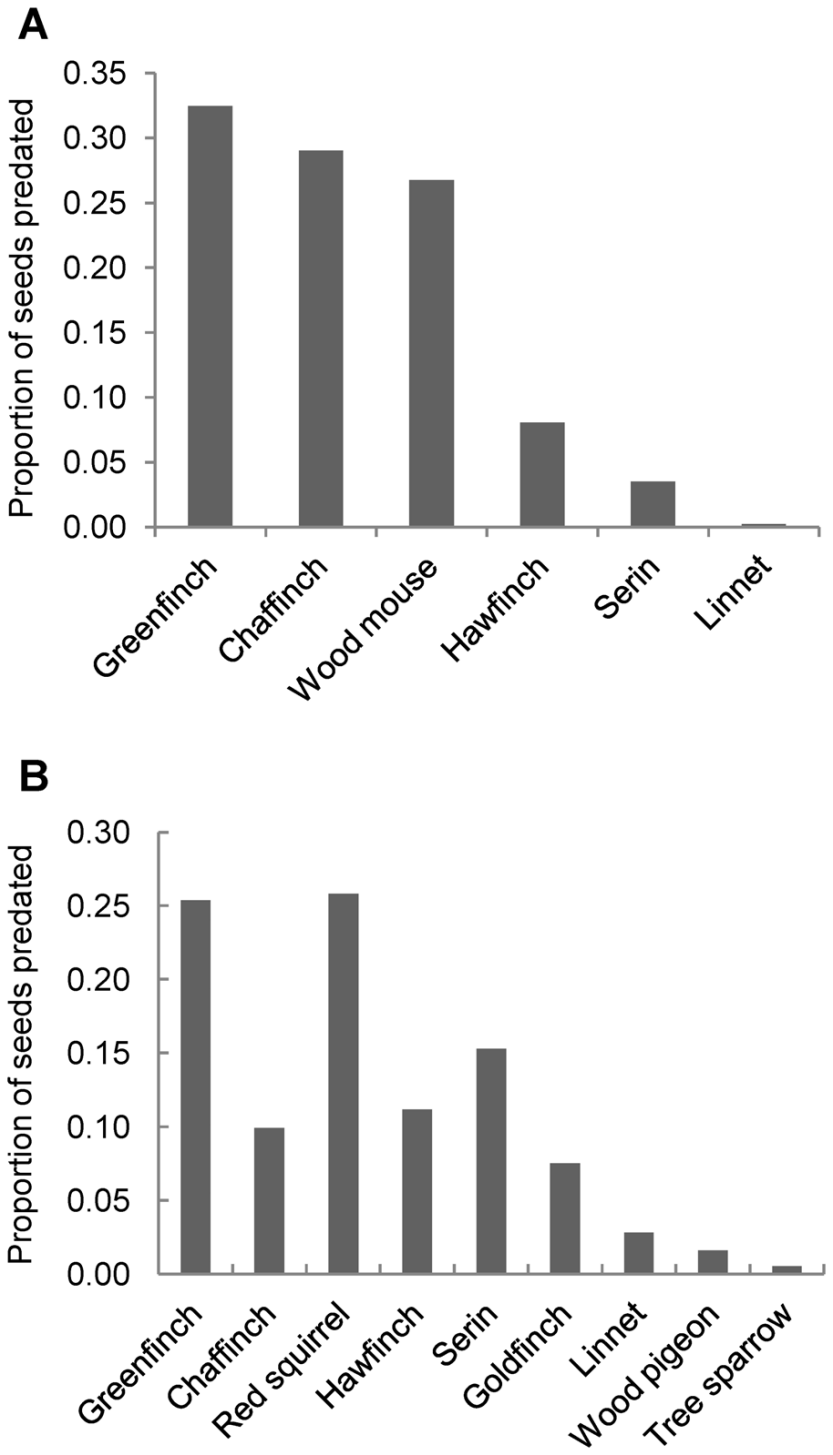
Proportion of samaras consumed by each animal species (*n*>5 contacts). a) on the ground (open microhabitat); b) in the tree. Greenfinch = *Carduelis chloris*; Chaffinch = *Fringilla coelebs*; Wood mice = *Apodemus sylvaticus*; Goldfinch = *Carduelis carduelis*; Serin = *Serinus serinus*; Linnet = *Carduelis cannabina*; Red squirrel = *Sciurus vulgaris*; Wood pigeon = *Columba palumbus*; Tree sparrow = *Passer montanus*.

We only obtained 9 recordings of animals removing or consuming samaras in the low branches of *Ulmus* trees. All of them were of chaffinches (*Fringilla coelebs*; *n* = 26 seeds). We obtained 281 video recordings of animals consuming elm samaras on the ground: four bird species in the family Fringillidae and the wood mouse (*Apodemus sylvaticus*) were the main seed predators there (Fig. 3A; *n* = 1280 seeds). Other species (number of recordings <5) were red squirrel (*Sciurus vulgaris*), wood pigeon (*Columba palumbus*) and linnet (*Carduelis cannabina*).

### Seed Predation in the Tree

In year 2010, we observed 1743 seeds eaten by arboreal seed predators (1.08 predated samaras per minute of observation; *n* = 235 observations) whereas in 2011 we only observed 6 samaras eaten (0.01 predated samaras per minute of observation; *n* = 2 observations). We found that red squirrel was the only diurnal mammal consuming elm seeds in the trees (Fig. 3B). Six bird species of the family Fringillidae were responsible for 97.1% of avian seed predation (*n* = 1256 seeds), whereas other bird species, mainly wood pigeons and tree sparrows (*Passer montanus*) only consumed 2.9% of the seeds (Fig. 3B).

### Bird Density Estimation

**Figure 4:**
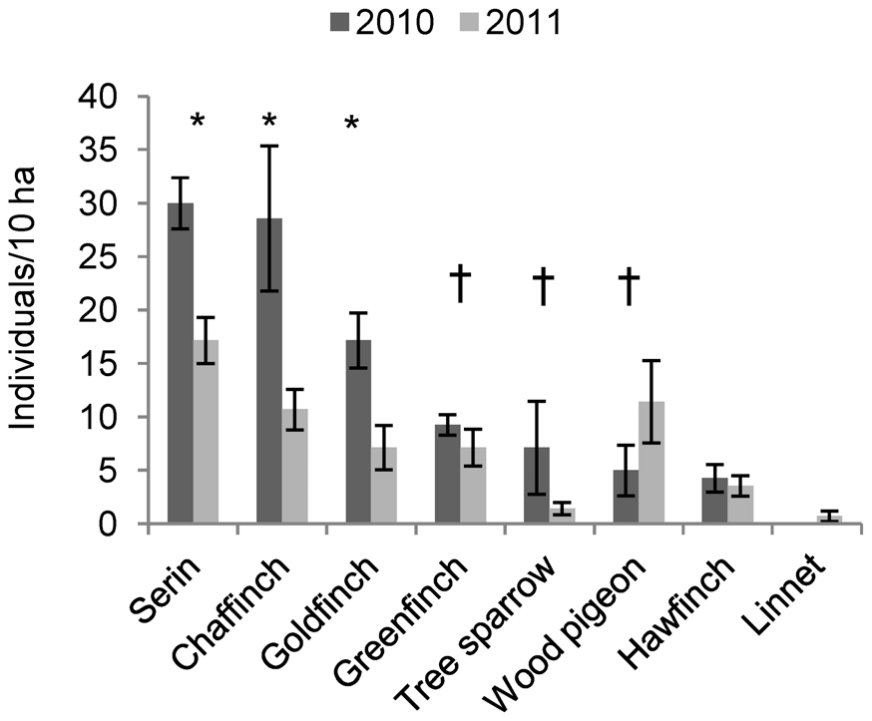
Density estimation of the main avian elm seed predators for two consecutive springs. Significant (*P*<0.05) and marginally significant differences (*P*<0.10) between years for each species are indicated by (*) and (†), respectively.

*Serinus serinus* and *Fringilla coelebs* showed the highest density estimates in both studied years (Fig. 4). We found a significantly higher bird abundance in 2010 compared to 2011 for serins (*Serinus serinus*), goldfinches (*Carduelis carduelis*) and chaffinches (*P*<0.05; Fig. 4). Greenfinches (*Carduelis chloris*) and tree sparrows also showed higher population densities in 2010 but differences were only marginally significant (*P*<0.10; Fig. 4). Similar population densities were obtained for hawfinches (*Coccothraustes coccothraustes*) and linnets whereas wood pigeon was the only bird species with lower density estimation in year 2010 (Fig. 4).

### Seed Predation on the Ground

**Figure 5:**
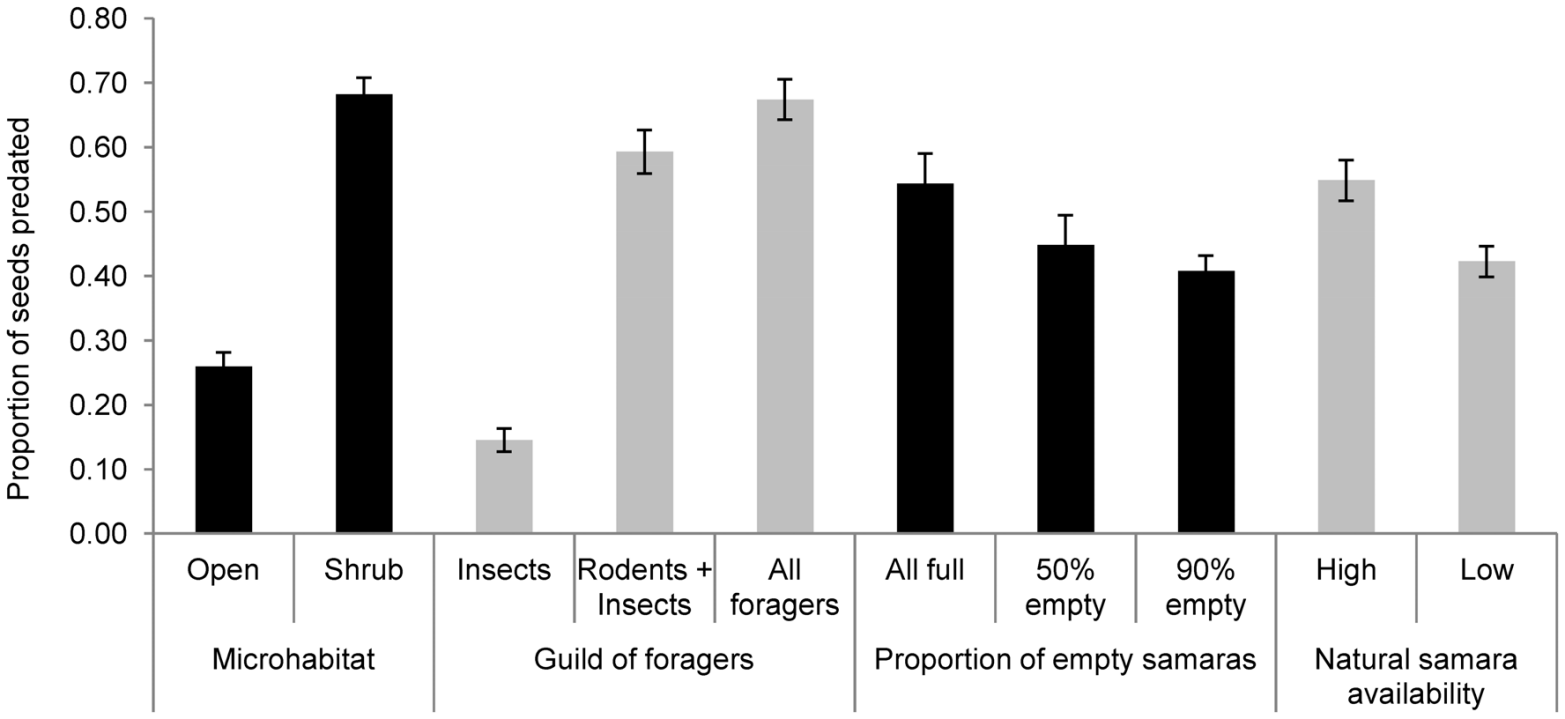
Proportion of seeds (full samaras) predated on the ground. Proportions are shown in relation to microhabitat of deposition, guild of foragers, proportion of empty samaras in the depot and natural samara availability.

**Figure 6:**
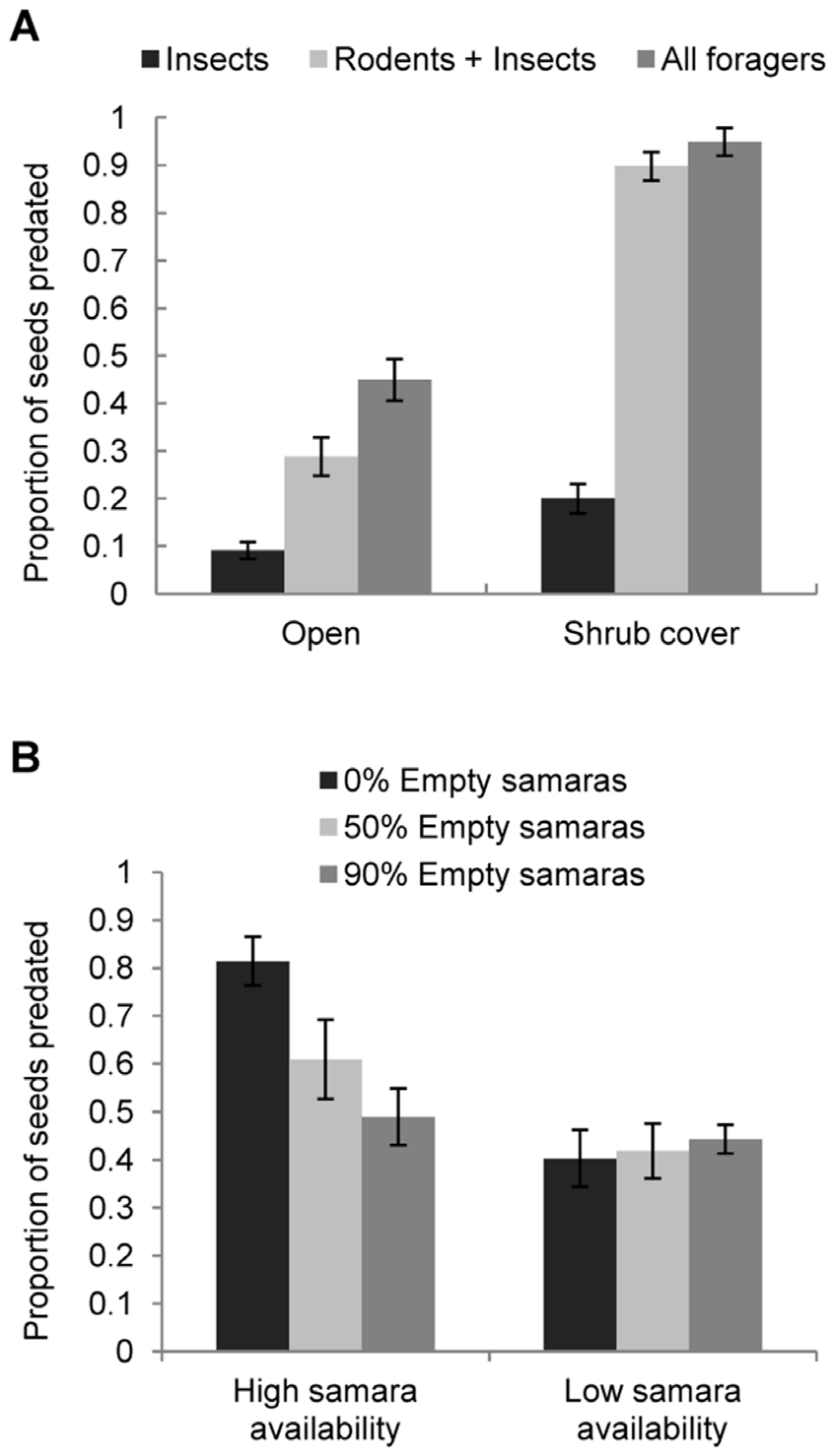
Main interactions found for the proportion of seeds (full samaras) predated on the ground. a) Relationship between microhabitat of samara deposition and guild of foragers; b) Relationship between natural samara availability and proportion of empty samaras. 0% empty samaras means that all samaras were full.

Proportion of seeds predated was significantly different across microhabitat of seed deposition, the guild of foragers involved and seed quality (Table 1; see Table S1 for the randomization approach). Proportionally less seeds were consumed in open microhabitats, within insect-only depots and when depots contained 90% empty samaras (Fig. 5). Additionally, we found a significant interaction between microhabitat of seed deposition and guild of foragers (Table 1 and Table S1), due to the fact that depots for rodents and depots for all foragers showed no differences in the proportion of seeds predated under shrub cover, whereas proportionally more seeds were consumed by all foragers in open microhabitats (Fig. 6A).

**Table 1:** Summary of the Wald χ^2^-test for the GLMM to analyze the factors affecting seed predation (proportion of full samaras predated in each depot).

	Seed predation		
Fixed effect	d.f.	*χ^2^*	*P*
Microhabitat (M)	**1**	**87.55**	**<0.0001**
Foragers (F)	**2**	**105.81**	**<0.0001**
Samara quality (Q)	**2**	**9.30**	**0.0095**
Samara availability (A)	**1**	**4.34**	**0.0371**
M×F	**2**	**17.53**	**0.0002**
Q×A	**2**	**19.77**	**<0.0001**

Microhabitat (M) refers to open vs. shrub cover; foragers (F) refers to depot type (exclosures) for different guild of seed foragers; samara quality (Q) is the proportion of empty samaras in the depot and samara availability (A) is the natural availability of samaras in the study area. Data for the best-fitting model (ΔAIC = 0); AIC = 282.4; Akaike weight = 0.69; Residual deviance = 258.4. Confidence intervals for the parameter estimates of this model and the randomized models are shown in Table S1 (bootstrapping).

Apparently, the proportion of seed predation was lower when seed availability was low (Table 1; Fig. 5). However, we must be cautious with the significance of this variable alone (*P* = 0.037; Table 1) since the randomization approach indicates some overlap between the observed and the randomized confidence intervals for this parameter estimates (Table S1). Nevertheless, samara availability showed a strong and significant interaction with seed quality in the proportion of seeds predated (Table 1 and Table S1). Thus, we found differences in the proportion of samaras predated across the different levels (proportions) of empty samaras only during high seed availability (Fig. 6B). Conversely, in years with low seed availability, we found no effect of different levels of empty samaras on the proportion of full samaras consumed (Fig. 6B).

## Discussion

### Relationship between Seed Availability and Seed Predation

We found that the number of pre-dispersal seed predators (bird visitations) as well as bird densities in the study area significantly decreased in a low seed production season. In addition, there was an apparently higher proportion of seed predation on the ground in seasons with higher seed availability (Fig. 5). This would contrast with multiple studies that report either no correlation [13], [18], [35], [36] or a negative relationship between seed crop size and seed predation [14], [26], [37]–[39]. However, few studies have shown a positive relationship between proportion of seed predation and seed availability [40], [41]. Other authors [42] also showed that birches (*Betula alleghaniensis* Britt.), when producing large seed crops, increased avian seed predation but reduced seed losses by invertebrates. Predator satiation may work for animals that have low mobility (e.g. small rodents and insects) [14]. However, in our case, proportion of post-dispersal seed predation by small rodents (wood mice) increased with seed availability, which could be interpreted against a possible support to predator satiation theory. Conversely, if we consider numbers of seeds and not proportions, it comes that, overall, in a mast event more seeds remain unconsumed, probably increasing plant fitness through a higher number of potential recruits. Another study [43] also showed that in mast years the rate of seed removal by rodents was higher, facilitating the hoarding of seeds, which is not necessarily contrary to predator satiation hypothesis since animals could become satiated at the time of seed recovery. In that way, masting would have a previous effect of higher removal, followed by a later satiation, which has been demonstrated to facilitate seed dispersal and eventual seed survival, especially when seeds are scattered-hoarded [43]. However, samaras are low-value seeds and, thus, are mostly eaten and rarely hoarded by wood mouse [29, Perea et al. pers. obs.].

### The Importance of Producing Empty Seeds

We found that production of empty seeds helped expand the presence of seeds in the tree, increasing considerably (2–3 fold) the overall seed drop period (Fig. 2C). This longer presence of seeds in the tree could reduce the attraction of highly mobile and more generalist predators such as birds and may satiate other less mobile animals (insects, rodents) through a higher synchronization in the fall of full seeds (Fig. 2C). This alternative would explain how production of empty seeds can serve to prolong the overall seed drop period and to exhibit greater variation in seed production (higher CV) and, thus, will help to cope with both, local specialist and more generalist (highly mobile) predators, in agreement with [44].

Surprisingly, empty seeds were not prematurely released despite their inability to produce new seedlings. The fact that empty seeds remain attached to the tree so long could be an adaptive strategy to reduce seed predation in the tree [13]. This ensures a simultaneous presence of both full and empty seeds, and, consequently, increases their effectiveness to deceive seed predators since animals completely rejected the empty-seeded fruits (no empty samaras were eaten). Moreover, we show that this pattern was similar for the three studied years and, thus, independent of the seed crop size. Here, we highlight that the cost of producing empty seeds is much lower than producing full seeds (in dry biomass and C, N, P allocation). This explains why producing and maintaining empty-seeded fruits in the tree is energetically efficient, by simply increasing the probability that full seeds escape predation.

In addition, we clearly distinguished between undeveloped samaras (aborted seeds), which were produced in a very low quantity and early in the season, and empty samaras (parthenocarpic), which were the most common type of infertility (Fig. 2A). We believe there is a need to distinguish parthenocarpic and undeveloped fruits not only morphologically but also in terms of energy cost. This distinction could help to understand why empty samaras (parthenocarpic fruits) remain attached to the tree so long in comparison to aborted seeds.

### Interaction between Seed Availability and the Proportion of Empty Seeds

Interestingly, we found a significant interaction between seed crop size and the proportion of empty seeds in relation to seed predation. To our knowledge this is the first study revealing that seed predation (proportion of full seeds eaten) decreases as the proportion of empty seeds increases but only in high crop sizes (Fig. 6B). However, in low seed production events, we found no effect of parthenocarpy on the predation rate of full samaras (Fig. 6B). This suggests that there could be a potential reproductive benefit in producing empty seeds in seasons with high seed availability but not in years with low seed production. Production of low number of seeds, independently of the seed type (full or empty), already reduced pre-dispersal seed predation, since low crops are unattractive to generalist predators, especially flocks of avian seed predators. For example, Shaw [45] reported that pigeons were not attracted to oak trees in light acorn crops. Other studies found that overall number of seeds consumed by predators (from the total fruit crop) was lower when there were a higher proportion of empty seeds [12], [18]. Here, we suggest that this effect of empty seeds reducing seed predation depends on crop size. Thus, in mast events, an extra production of empty seeds among many full will not considerably increase attraction of generalist foragers (already attracted by a mast crop), but will better deceive the foragers and increase their search and handling costs (effort and time). The interesting part of deceiving predators is to know what is the ideal proportion (the minimal cost for the plant) to successfully deceive the predators and maximize seed survival. In this study we found that a proportion of 50% empty seeds significantly reduced seed predation by a 26% (Fig. 6B, left side). Although an increase in the percentage of empty samaras up to 90% raised those values (from 26% to 39% in seed predation; Fig. 6B), differences were not significant between 50% and 90% ES, indicating that a very high rate of empty samaras is probably not worthwhile in terms of reproduction efficiency. Further studies should estimate the most adequate proportion of empty-seeded fruits to minimize seed predation and maximize reproduction efficiency. It could be that the value of 50% empty-seeded fruits is too high, and other values found for fleshy-fruited plants such as 30% for *Pistacia lentiscus* [13] could be more appropriate. Nevertheless, it seems that variability in the production of empty samaras (25±24% in this study for individual trees) could work better than a fixed value to deceive the predators and reduce predation, as occurs in the masting phenomenon, where predator-dispersed plants have high coefficients of variation in seed production, consistent with the idea of escaping seed predation more easily [41].

### Origin of Parthenocarpic Fruits

The reasons why elm trees produce a high proportion of parthenocarpic fruits are still unclear, but the massive production of empty seeds regardless the seed crop, together with the late dispersal of these unviable seeds, suggest a possible adaptation to reduce seed predation. It has been proved that seed infertility in some wind-dispersed trees is mostly due to maternal genotype (genetic load) and not to self-pollination [47]. However, we need further studies to corroborate whether seed predation is acting upon genetic load, and whether parthenocarpy could be an adaptation to reduce seed predation in wind-dispersed trees. A heterogeneous distribution of empty samaras among branches or inflorescences will reinforce the adaptive hypothesis, whereas the presence of complete infertile individuals, producing mostly empty-seeded fruits will help to discard this hypothesis. Our results already revealed that there are no complete sterile trees. In fact, we found a rather high variability in the production of empty seeds for the same individuals in two consecutive years. Additionally, the inter-individual variability in the proportion of empty seeds was greater in the mast year and lower in non-mast events. This high variability (especially in mast years), together with the general unpredictability in the production of empty seeds, reinforce the idea of deceiving the predators more easily. The most plausible explanation for the variable production of empty-seeded fruits would be the variable weather conditions affecting, for instance, pollination. However, parthenocarpy was very common in the three studied years and highly variable within the population and within each individual (more variable that expected for climatic factors) and, thus, we suggest that weather alone could not be responsible for the highly variable parthenocarpy. This agrees with the fact that masting has been also considered an adaptive strategy functioning under the weather influence [46].

### Open Microsites Facilitate Seed Survival

This study also reveals an important post-dispersal predation of elm seeds, mainly by birds and mammals. We found strong differences in the proportion of seeds predated depending on the microsite where samaras were deposited. Thus, rodents mostly preyed upon or removed seeds when samaras were located under shrub cover, their preferred microhabitat [11]. Unlike rodents, Fringillidae birds consumed higher number of seeds in open areas, possibly because birds search for seeds visually and not olfactorilly [48]. Additionally, we observed how finches feel safer in open areas where they can easily fly away, probably as a strategy to escape predation [49]. In general, rodents were the main post-dispersal seed predators in line with other studies [29]. Surprisingly, however, Fringillidae birds (mainly *C. chloris* and *F. coelebs*) were important seed predators on the ground according to the video recordings obtained. This contrasts with other studies [24], [29] and reveals that birds can be important post-dispersal predators of samaras, increasing their relative contribution as the proportion of open microhabitats increases. Thus, a system with abundant open microhabitats will decrease the overall ground seed predation by reducing seed predation by rodents (the main ground foragers), although there will be an increase in avian seed predation. However, in low crop sizes we found that density of avian seed predators decreased (Fig. 3), probably because finches moved to areas richer in seeds. Granivorous birds have greater mobility than small rodents and, therefore, can move more easily among patches and escape to low crops. As a result, in years with low samara production, granivorous birds will prey upon very few seeds and will be much less detrimental than rodents. For these low crop events, open areas will be especially important to facilitate seed survival and plant recruitment since riparian *Ulmus* are light demanding species [50].

## Supporting Information

Table S1 **Summary of the parameter estimates (95% confidence interval) for the randomized models (bootstrapping).** The number of iterations was 2000 in all cases. Random samples were of N = 540 with replacement.
(DOCX)
