# Correction: Empty Seeds Are Not Always Bad: Simultaneous Effect of Seed Emptiness and Masting on Animal Seed Predation

**DOI:** 10.1371/annotation/4690ecf8-cf07-4e28-9d81-855f8113fef7

**Published:** 2013-09-19

**Authors:** Ramón Perea, Martin Venturas, Luis Gil

The second author (MV) was incorrectly omitted from the list of authors who "Conceived and designed the experiments." 

