# Supplementary material for: Empty Seeds Are Not Always Bad: Simultaneous Effect of Seed Emptiness and Masting on Animal Seed Predation
Source: PLoS One. 2013 Jun 11;8(6):e65573. doi: 10.1371/journal.pone.0065573 (PMC3679161; doi:10.1371/journal.pone.0065573)
Supplement: Table S1 — Summary of the parameter estimates (95% confidence interval) for the randomized models (bootstrapping). The number of iterations was 2000 in all cases. Random samples were of N = 540 with replacement. (DOCX) [file pone.0065573.s001.docx]

| Parameter | Confidence intervals for the best-fitting model (ΔAIC=0) | | | Confidence intervals when randomizing the response variable | | Confidence intervals when randomizing the variable seed availability | | Confidence intervals when randomizing the variable seed quality | |
| --- | --- | --- | --- | --- | --- | --- | --- | --- | --- |
|  | Observed Value | Low95 | Upp95 | Low95 | Upp95 | Low95 | Upp95 | Low95 | Upp95 |
| Intercept | -2.9175 | -3.7528 | -2.3062 | -0.5610 | 0.3012 | -3.4603 | -2.1051 | -2.9678 | -1.6268 |
| Microhabitat (M) | 0.9710 | 0.5053 | 1.5105 | -0.5604 | 0.5421 | 0.4445 | 1.5731 | 0.3959 | 1.5386 |
| Foragers (F).f2 | 1.5581 | 0.9827 | 2.1849 | -0.5218 | 0.5361 | 0.9188 | 2.1343 | 0.9150 | 2.0981 |
| Foragers (F).f3 | 2.1325 | 1.6532 | 2.7410 | -0.5194 | 0.5406 | 1.4622 | 2.6349 | 1.4712 | 2.6243 |
| Seed Quality (Q).f2 | 1.5656 | 0.7966 | 2.5100 | -0.7404 | 0.7336 | -0.0967 | 1.2273 | **-0.7945** | **0.7116** |
| Seed Quality (Q).f3 | 2.7233 | 2.1423 | 3.5731 | -0.7812 | 0.7207 | 0.0359 | 1.6344 | **-0.7887** | **0.7064** |
| Seed Availability (A) | 0.2220 | -0.2884 | 0.7182 | -0.3677 | 0.3872 | **-0.4659** | **0.4674** | -0.8350 | 0.0668 |
| M.f2 × F.f2 | 2.4299 | 1.6695 | 3.5671 | -0.7895 | 0.7958 | 1.5202 | 3.3928 | 1.4327 | 3.4000 |
| M.f2 ×F.f3 | 2.6145 | 1.7780 | 3.8935 | -0.7913 | 0.7516 | 1.7132 | 3.9959 | 1.6743 | 3.8861 |
| Q.f2 × A.f2 | -1.5641 | -2.6764 | -0.5759 | -0.9072 | 0.9201 | **-0.9557** | **0.9769** | **-1.0797** | **1.0304** |
| Q.f3 × A.f2 | -3.0422 | -4.3045 | -2.1448 | -0.9139 | 0.9391 | **-1.0851** | **1.0865** | **-0.9910** | **1.0782** |
